# PEM-PCA: A Parallel Expectation-Maximization PCA Face Recognition Architecture

**DOI:** 10.1155/2014/468176

**Published:** 2014-04-15

**Authors:** Kanokmon Rujirakul, Chakchai So-In, Banchar Arnonkijpanich

**Affiliations:** ^1^Applied Network Technology (ANT) Laboratory, Department of Computer Science, Faculty of Science, Khon Kaen University, Khon Kaen, Thailand; ^2^Department of Mathematics, Faculty of Science, Khon Kaen University, Khon Kaen, Thailand

## Abstract

Principal component analysis or PCA has been traditionally used as one of the feature extraction techniques in face recognition systems yielding high accuracy when requiring a small number of features. However, the covariance matrix and eigenvalue decomposition stages cause high computational complexity, especially for a large database. Thus, this research presents an alternative approach utilizing an Expectation-Maximization algorithm to reduce the determinant matrix manipulation resulting in the reduction of the stages' complexity. To improve the computational time, a novel parallel architecture was employed to utilize the benefits of parallelization of matrix computation during feature extraction and classification stages including parallel preprocessing, and their combinations, so-called a Parallel Expectation-Maximization PCA architecture. Comparing to a traditional PCA and its derivatives, the results indicate lower complexity with an insignificant difference in recognition precision leading to high speed face recognition systems, that is, the speed-up over nine and three times over PCA and Parallel PCA.

## 1. Introduction

Face recognition has recently brought the extensive attention to the society for both research and commercial, especially when several applications have been practically adopted in several areas, for example, human biometrics, pattern recognitions, and computer visions, within various practical usages such as access controls, human identifications, robotics, crowd surveillances, and criminal forensics [1]. In fact, one of the computational techniques used to automatically identify and/or verify a human face is facial recognition systems. Facial recognition systems focus on the unanimated image of a human's face. The image can be retrieved either from a digital picture snapshot or a video frame. Generally, there are two subsystems: face detection [2] and face recognition [3, 4]. The first approach is used to identify the face position before specifically distinguishing the face identity in the recognition stage. There are a number of researches proposed to achieve high precision of the first subsystem [5–7]. However, although some approaches have been introduced to resolve the second subsystem, several issues which are among research community interests still remain. Thus, in this research, the focus is on the latter.

Consider the face recognition stage. There are many approaches used to enhance a recognition precision, one of them is to compare the properly selected facial features' images to their facial database [8]. In general, a common image-based face recognition method with feature-based extraction can be divided into two categories: appearance-based and model-based approaches [9]. Each of which has its own distinctive characteristic; for instance, the first scheme, which applies the concept of transformed face data to a face space analysis problem without using the human knowledge, is designed to support the images with low resolution and/or poor quality. For the second approach, each standing point of the actual face structure including face variation is formed as the face feature model, normally required an interaction with the human. Aside from a specific face model, that is, expression and position human images, the first approach yields high accuracy widely used in the traditional image-based face recognition. Thus, many proposals adopted the appearance-based approaches, for example, Principal Component Analysis (PCA), Independent Component Analysis (ICA), Linear Discriminant Analysis (LDA), Kernel Principal Component Analysis (KPCA), and ISOMAP [9].

Consider those approaches; however, PCA and its derivatives are commonly used due to several distinctive features. For example, PCA is an optimal linear scheme in terms of mean squared error for compressing a set of high dimensional vectors into a set of lower dimensional vectors. In addition, the model parameters used in PCA can be directly computed from the data without additional processing steps. Moreover, compression and decompression operation complexity is given to the model parameters; in other words, PCA only requires matrix multiplication. Most importantly, PCA requires less number of features, which provide the nonreduction of precision quality, and these advantages result in high recognition accuracy even with a small data set [4]. Although PCA expresses several distinctive outcomes, one of the drawbacks is over a high complexity due to larger matrix operation. A large amount of memory is also required since the memory requirement grows with the image quality, high resolution, and the number of training images used for image matching or classification [10, 11].

With PCA derivations, for years, there are many attempts to overcome the drawbacks of a traditional PCA to enhance the performance of PCA recognition scheme, for example, a symmetrical PCA and two-dimensional PCA [9]. Some techniques were involved in finding alternative approaches to lessen the computational complexity of PCA, for example, applying QR decomposition instead of singular value decomposition (SVD) [11]. Nevertheless, a few of them focus on the feasibility to absorb the high computational stage, matrix manipulation, and to optimize the computational stages, especially utilizing the parallelism to enhance the speed-up of PCA. More details will be discussed in the related work section.

With a high computational task, one of the probable approaches to lessen the serial constraint is the usage of parallelism concepts. Recently, with a rapid increase of computer chips and advances in integrated circuit technology results in affordable multicore CPUs and enhanced parallelization techniques [12], which have risen for a specific computational task, becoming a practical solution. As a result, this research has also investigated a possibility to improve the face recognition system, and so our contribution lies in two folds: first, using Expectation-Maximization (EM) instead of PCA covariance matrix computation for face feature extractions and, second, a novel parallel architecture for enhancing the speed-up on matrix computational operations over our first optimization called EM-PCA including the enhancement over face classification parallelization. The contribution forms novel parallel face recognition architecture, so-called a Parallel Expectation-Maximization PCA architecture (PEM-PCA).

This research paper is organized as follows. In Section 2, background of face recognition systems is briefly revisited including PCA and its limitation. Then, in Section 3, closely related works of a traditional PCA, PCA derivations' optimizations, and a parallel system for PCA face recognitions are comparatively surveyed.  Section 4 presents the parallel architecture proposal including the first PCA optimization (EM-PCA) and the second parallel face recognition architecture in three substages (PEM-PCA). After that, in Section 5, our comparative performance of various proposals is discussed. Finally, the conclusions and future work are drawn in Section 6.

## 2. Background

**Figure 1:**
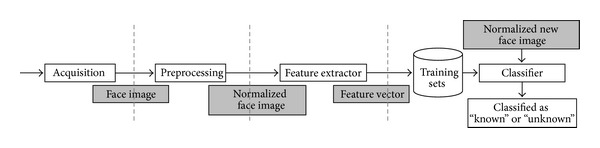
Face recognition system.

In general, a face recognition system consists of four components shown in Figure 1. These components are acquisition, preprocessing, feature extractor, and classifier [13]. Additionally, in order to recognize the face identity, there are two main processes: training and testing. For training process, given an image acquisition step from various inputs, for example, captured photos, scanned images, and video frames (acquisition), face images are fed as inputs into the preprocessing step, for example, histogram equalization, background removal, pose adjustment, image size normalization, gray-scale transformation, median filtering, high-pass filtering, translation and rotational normalizations, and illumination normalization, in order to generate normalized face images. Then, the system extracts the main features of the images (feature extractor) resulting in feature vectors stored in a training set.

The testing process is similar to the training process but with fewer steps. The testing image will be processed in order to generate proper normalized face images which are ready for face image classifier (classifier) in order to figure out the least feature matching distance between testing and trained features. Normally, there are several techniques of feature extractor as well as classifier, that is, PCA, ICA, and LDA, as face image matching, for example, Euclidian distance (ED), support vector machine (SVM), and K-nearest neighbor [1]. It should be noted that PCA and ED are commonly used due to distinctive characteristics, that is, low complexity yielding high classification speed [14, 15].

**Figure 2:**
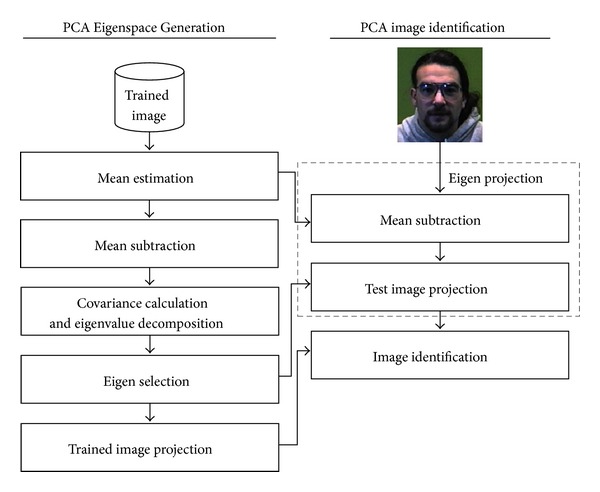
PCA Eigenspace Generation and PCA image identification.

Specifically consider PCA [16], one of the well-known approaches for face recognition systems. PCA applies a line transformation technique over a sample image to reduce the set of large images' variances and then projects their variances into coordinate axis. In other words, the goal of PCA is to decrease the high dimensional image data space into low dimensional feature space [17].  Figure 2 shows an overview of PCA face recognition system. Here, there are two main components: PCA Eigenspace Generation and PCA image identification. Compared to Figure 1, these two components correspond to two modules: feature extractor and classifier. Note that normally, before processing the PCA face recognition stages, it requires a preprocessing step as shown in Figure 1, and one of the necessities is to load the input images into an initial matrix, each of which will be converted from RGB to grayscale and then reshaped into a common size. This matrix is also generally normalized in order to reduce the data size leading to the reduction of time complexity [13].

1. PCA Eigenspace Generation: there are five submodules in PCA feature extraction computation as follows: (1) estimating the mean vector of trained images; (2) centering the input data around the mean vector by finding the difference between the input image and images' mean; (3) performing the covariance matrix calculation and then applying SVD over the covariance matrix to obtain the eigenvectors and the eigenvalues; (4) sorting the eigenvector in descending order and then selecting nonzero eigenvalues; and finally (5) projecting training images by calculating the dot product between the trained image and the ordered eigenvectors.
2. PCA image identification: there are three submodules as follows: (1) subtracting the testing image by mean vector; (2) performing Eigenspace projection by executing dot-product computation; (3) projecting the testing image and making a comparison between training and testing images to retrieve the closet distance.

As discussed previously, applying PCA for face recognition incurs several advantages; however, there are some limitations; for instance, PCA involves a complex mathematical procedure due to a transformation from a large number of correlated variables to a smaller number of uncorrelated ones. Thus, in particular, a high resolution image in addition to a large number of images produces high computational complexity, especially during the matrix manipulation, that is, multiplication, transpose, and division, among high dimensional vectors [18].

## 3. Related Work

There are many face recognition proposals employing PCA [9]. Shamna et al. and Zhao et al. indicated that most of them used PCA for different purposes and obtained several distinctive features, for example, less memory requirement and simple computation complexity [5–7, 15, 19–21]. The results of the survey brought about various PCA derivations including increasing the recognition rate. For example, in 2010, Gumus et al. [22] applied a hybrid approach over PCA and wavelets to extract feature resulting in higher recognition rate. Recently, some researches have also improved the recognition rate; for instance, Bansal and Chawla [23], in 2013, have proposed normalized principal component analysis (NPCA) which normalized images to remove the lightening variations and background effects by applying SVD instead of eigenvalue decomposition.

Furthermore, in the same year, Yue [24] proposed to use a radial basis function to construct a kernel matrix by computing the distance of two different vectors calculated by the parameter of 2-norm exponential and then applying a cosine distance to calculate the matching distance leading to higher recognition rate over a traditional PCA. Similarly, Min et al. [17] introduced a two-dimensional concept for PCA (2DPCA) for face feature extraction to maintain the recognition rate but with lower computational recognition time. It should be noted that only a few proposals investigated a computational time complexity.

Consider computational time complexity. In 2011, Chen et al. [25] proposed a local facial feature framework for still image and video-based face recognition leveraging Feature Averaging (FA), Mutual Subspace Method (MSM), Manifold to Manifold Distance (MMD), and Affine Hull Method (AHM) resulting in high speed processing, especially for CCTV surveillance, but with the limitation of lower recognition rate, that is, only 90%. Similarly, however, Pereira et al. [26] proposed a technique to reduce face dimensions called class-modular image principal component analysis (CMIPCA) to extract local and global information to reduce illumination effects, face expressions, and head-pos changes resulting in speed-up over PCA. Recently, W. Wang and W. Wang [16] have also applied K-L transformation for feature extraction to speed-up recognition rate but still maintaining recognition precision.

Specifically consider recognition stage complexity. Roweis [28] generally discussed a possibility to use Expectation-Maximization or EM algorithm for PCA to resolve the covariance computation time of PCA-based problem in general. This technique does not require a sample covariance computation, and so, a complexity is substantially reduced when compared to a traditional PCA. In addition, Ahn and Oh [29] proposed a constrained EM algorithm to enhance the performance of PCA to resolve the actual principal components extracting the problem using a couple probability model derived from single-standard factor analysis models with isotropic noise structure.

Moreover, in 2010, Chan and Tsai [30] applied EM algorithm over PCA to identity emotion of facial animations, but not for realistic human faces. Two years later, Tsai [31] showed an application of dimensionality reduction techniques for computer facial animation in various techniques, for example, PCA, EM-PCA, Multidimensional Scaling, and Locally Linear Embedding. In addition, in 2012, Reel et al. [32] used EM-PCA to recognize medical images, for the purpose of computational complexity reduction. A few months later, they [33] also proposed a similar technique involving an initial known transformation of predefined axes' translation and rotation, and these techniques resulted in a reduction of inherent dimensionality problem leading to lower computational complexity. Aiming at distinctive advantages of EM, note that the EM derivation is our promising technique to hybrid with PCA for human face recognition.

In order to overcome the major limitations of single core processing, one of the promising approaches to speed up a computation is parallelism. Several parallel architectures including parallel algorithms and machines have been investigated. Most of parallel face recognition systems only applied computer hardware architectures; for example, each individual computer system is used to run each individual image or a subset of face images. Additionally, recently in 2013, Cavalcanti et al. [34] proposed a novel method called (weighted) Modular Image PCA by dividing a single image into different modules to individually recognize human face to reduce computational complexity.

Previously, in 2003, Jiang et al. [35] proposed a distributed parallel system for face recognition by dividing trained face databases into five subdatabases feeding into each individual computer hardware system and then performed an individual face recognition algorithm individually in parallel over TCP/IP socket communication, and after that, the recognition feedbacks are sent back for making a final decision at the master. Similarly, Liu and Su [36] modified a distributed system to support parallel retrieval virtual machines by allowing multivirtual machines for each slave to run individual face recognition algorithms.

To improve recognition accuracy, in 2005, Meng et al. [1] applied a parallel matching algorithm with a multimodal part face technique. This technique uses five main face parts on each face that is bare face based on a principal component analysis and then used for recognition process in each individual computer hardware system enabling MMX technology to speed up the matching process. In addition, in 2007, Huiyuan et al. [18] proposed a division of eigenblock in equal block size and then performed PCA face recognition in distributed manners. This process can enhance the speed-up; however, the accuracy is still under consideration.

Due to the advances of multicore-processors within a single computer system, Wang et al. [37] proposed a parallel face analysis platform which basically used two-level parallel computing architecture. The first level is assigned to each individual core for recognition purpose by dividing testing and training images in parallel, and the second level is only to perform the final decision from the results of recognition processes. In 2010, Numaan and Sibi [38] also discussed a possibility of parallelizing and optimizing PCA with eigenfaces; however, there is a limitation of memory size.

Notice that all of the approaches discussed above can achieve sorts of highest degree of parallelisms by only performing an individual face recognition algorithm either in multivirtual machines or multicore-processing with the key limitation on the number of CPU cores, and so, in general, these approaches do not utilize the parallelism in each face recognition stage in order to achieve higher degree of parallelisms, and these are our main focus in this research to propose a parallel architecture utilizing the parallelism of face recognition stage.

Aside from the recognition stage, the other two stages, preprocessing and classification phases, are also important. For example, consider the first phase. Zhang [20] introduced wavelet transform, discrete cosine transform, and color normalization, as preprocessing methods in face recognition to achieve better recognition precision. Recently, Kavitha et al. [39] have also introduced color space transformation (CST) with fractional Fourier transform (FFT) and local binary pattern (BP) to increase recognition rate, both of which lack including the investigation of stage parallelism to further speed-up.

Consider the classification stage. Many approaches are introduced, for example, ED, Manhattan distance, Mahalanobis distance, nearest neighbor, and SVM [4, 9, 40]. For example, in 2013, I. Melnykov and V. Melnykov [41] proposed the use of Mahalanobis distance for K-mean algorithm to improve the performance when covariance matrices are not properly initialized, but with the increase of computational complexity. Moreover, soft computing-based approaches, that is, neural networks, are also used to compute the matching distance; for instance, Yue [24] used nearest neighbor methods to compute the matching distance to improve the classification precision. Zhou et al. [14] also proposed a combination of PCA and LDA for image reconstruction to increase recognition rate and then be classified with SVM. Similarly, Gumus et al. [22] also applied SVM during classification steps resulting in higher recognition rate but higher computational time.

It should be noted that most of the face recognition systems have applied ED for face classification [2–4, 9, 34] to achieve simplification and to yield acceptable classification precision. To emphasize a feasibility of this technique, in 2003, Draper et al. [15] compared Manhattan distance, ED, and Mahalanobis distance over PCA and then found out that ED have the highest accuracy rate. In addition, recently, in 2013, Moon and Pan [42] compared Manhattan distance, ED, cosine similarity, and Mahalanobis distance over LDA face recognition in which the results lead to an outstanding performance of ED. In addition, to further speed up the computational time, Li et al. [43] proposed ED for matrix calculation on a large dataset using multiprocessing, which applied a traditional parallel matrix operation.

To sum up, as discussed above, PCA yields high face recognition precision together with several derivations; however, our proposals investigated enclosing Expectation-Maximization (EM) algorithm into PCA to reduce a computational time complexity during covariance calculation. To further enhance the speed-up of the recognition rate, although many proposals focus on the parallelism utilization, our proposal deals with individual stage parallelisms during matrix manipulation of our first enhancement by rearranging the matrix manipulation including determinant and orthogonalization processes. Last but not least, the optimization over parallel classification technique was also investigated. These three combinations lead to a parallel architecture for face recognition called Parallel Expectation-Maximization PCA architecture (PEM-PCA).

## 4. Parallel Expectation-Maximization PCA Face Recognition Architecture (PEM-PCA)

**Figure 3:**
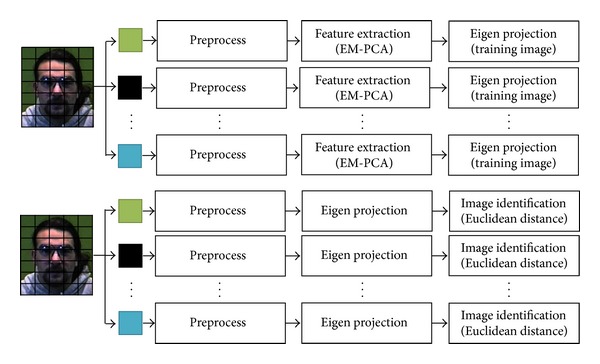
A Parallel EM-PCA Architecture (PEM-PCA).

An overall architecture of Parallel Expectation-Maximization PCA (PEM-PCA) generally consists of three parts: parallel face preprocessing, parallel face feature extraction, and parallel face classification. To illustrate the detailed system, Figure 3 shows both training and testing processes of PEM-PCA in comparison to a traditional face recognition system, as shown in Figure 1, excluding an acquisition stage. In this architecture, both training and testing processes will be performed in parallel. In general, initially, an input image is divided into pixels and then executed in parallel, that is, one pixel per thread. During a high matrix computation complexity, a manipulation process is performed in parallel by computing one element of a result matrix per thread. It is observed that the parallel efficacy depends upon a number of cores and relationships between thread and core usages. For example, if there are twenty processes on eight cores, basically, it performs the first eight processes followed by the latter once completed iteratively.

### 4.1. Parallel Face Preprocessing

**Figure 4:**
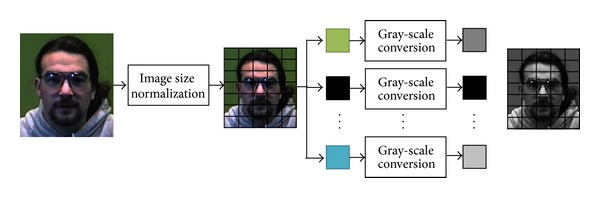
Parallel preprocessing.

In general, preprocessing is one of the major stages used to reduce computational complexity as well as increase recognition precision for noise reduction. Although there are several preprocessing techniques, briefly stated in related work section, here, a necessary parallel method is considered to aid algorithm efficiency, that is, gray-scale conversion. Regarding our previous experiment, the recognition precision between color and gray-scale images is not significantly different but with the increase of computational time complexity [27]. Thus, in our proposed architecture, our focus is to parallelly perform gray-scale conversions once the input images are shaped into the same size, that is, 180 × 200 pixels in both training and testing processes as shown in Figure 4. Note that other probable preprocessing techniques can also be fit in this architecture with paralleled modification.

### 4.2. Parallel Face Feature Extraction

As stated in related works, several enhanced proposals over PCA for reducing a number of dimensions have been introduced; however, some issues are to resolve, for example, outlier and noise reductions, leading to lower accuracy, and computational complexity. Thus, here, to lessen the limitation, our first proposal is to apply Expectation-Maximization (EM) to figure out the maximum likelihood to estimate proper parameters derived from the covariance computational step [28] called EM-PCA. Then, to speed up the recognition rate, several simplified matrix manipulation techniques are to be proposed called Parallel EM-PCA (PEM-PCA).

#### 4.2.1. Expectation-Maximization PCA Face Recognition (EM-PCA)

**Figure 5:**
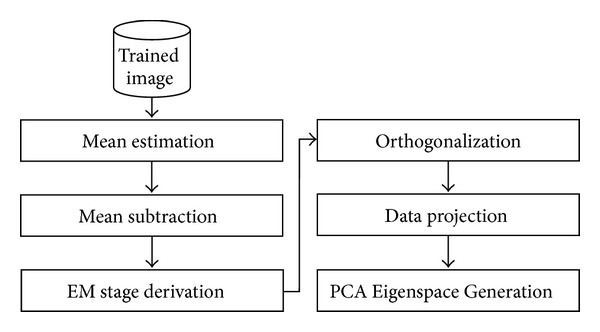
EM-PCA face recognition stage.

To enhance face recognition rate, Figure 5 illustrates our hybrid EM-PCA face recognition scheme when applying EM steps [44] instead of the covariance computational stage over a traditional PCA face recognition, stated in the second module of the first component as shown in Figure 2. Here, there are four substages: EM-Step derivation, orthogonalization, data projection, and PCA Eigenspace Generation. Before moving to the these stages, there are two more substages for preprocessing: mean (vector) estimation, *μ*, including centralized input data acquisition, $\hat{X}$, and mean subtraction used to compute the difference between the input data and mean. Notice that, with EM-PCA, only a few eigenvectors and eigenvalues are required to be extracted from a large amount of high dimensional data set. In addition, the covariance structure of an observed *p*-dimensional variable can be captured with the operation less than *p*(*p* + 1)/2 dimensions compared to *p* ^2^ of a traditional PCA with full covariance matrix computation.

(1) EM-Step derivation: given eigenvector matrix *U*, this process is used to estimate input parameters *U* for the next stage (orthogonalization). Here, there are two steps called E-Step and M-Step, illustrated in (1) and (2), respectively. Note that EM-Step will be repeatedly performed until the change,* epsilon *(*ε*), of the difference between variances is equal or less than a specific threshold value. It should be also noted that, before entering this step, the eigenvector will be randomly selected as the input:
  (1) $$\begin{array}{c} A={\left(U^{T}U\right)}^{-1}U^{T}\hat{X}, \end{array}$$
  (2) $$\begin{array}{c} U=\hat{X}A^{T}{\left(AA^{T}\right)}^{-1}. \end{array}$$
(2) Orthogonalization: at this stage, Gram-Schmidt Orthonormalization [32] was performed. In general, this process started by normalizing the first vector and then iteratively transformed the remaining vectors into weighted normalized vectors. Note that the detailed description corresponding to our proposed parallel algorithm will be discussed in next section.
(3) Data projection: at this stage, the input vector is projected into a transpose of M-Step matrix manipulation, and then it is multiplied to the result from mean subtraction step.
(4) PCA Eigenspace Generation: the final stage is performed as a final round using a traditional PCA: mean estimation, mean subtraction, covariance computation and SVD, eigenvalue selection, and trained image projection, respectively, as previously shown in Figure 2.

Consider algorithm complexity. As stated in Algorithm 1, EM-PCA complexity is in order of *O*(*knp*) versus *O*(*np* ^2^) for covariance computation used in the traditional PCA face recognition where *k* is the number of leading trained eigenvectors (extracted component). It should be noted that the degree of accuracy of EM-PCA will be also based on the selection criteria of number of eigenvectors and* epsilon *(*ε*) with a time complexity trade-off.

#### 4.2.2. Parallel Expectation-Maximization PCA Face Recognition (PEM-PCA)

To further enhance the speed-up, we propose PEM-PCA which introduces the parallelism in each computational stage. During EM-PCA face recognition, based on our observation, there are four different fundamental matrix manipulations: multiplication (matrix/constant), division (constant), transpose, and subtraction (matrix), each of which can utilize the parallelism depending on its distinctive characteristic. In addition to these four, three extra processes, determinant, cofactor, and orthogonalization, can also be performed in parallel in our architecture.

For example, for parallel matrix multiplication with 4 × 4 dimension, here, a number of threads can be divided into four parts in each dimension (*A*_1,*j*_ × *B*_*k*,1_) or sixteen threads (*A*_*i*,*j*_ × *B*_*k*,*m*_) to achieve high degree of parallelism. Nevertheless, there is a trade-off over task distribution and self-contained computational complexity considering a communication process cost. Similarly to achieve parallel matrix computation in each process of EM-PCA face recognition, the degree of parallelism is based on the dimension of matrix versus a number of computational threads supported. In general, each computational step is parameterized by an iteration value and applies with a work-stealing algorithm, and so the runtime process may reassign a set of iterations to other available threads, if any.

As discussed previously, our proposal is to hybrid those two techniques over a traditional PCA (Expectation-Maximization and parallelism). Essentially, each distinctive matrix manipulation is to investigate the degree of parallelism, that is, during subtraction, multiplication, division, transpose, determinant, cofactor, and orthogonal, stated in Algorithm 1, where the parallel algorithm structure is generated as proposed by Blelloch and Maggs [45].

(*1) Parallel Subtraction.* The subtraction of dimensional matrix can be performed in parallel to calculate the difference of each element at the same position stated in (3), and the algorithm of matrix subtraction is illustrated in Algorithm 2:

(3) $$\begin{array}{c} c\left[i,j\right]=a\left[i,j\right]-b\left[i,j\right]. \end{array}$$

(*2) Parallel Multiplication.* There are two types of matrix multiplication used in our proposal either matrix multiplication by matrix or by constant number. Consider the first multiplication with matrix. The value of each element in result matrix can be computed by employing (4), (5), and (6), respectively. These equations are independent, and thus, the computation can be performed in parallel as shown in Algorithm 2. The row dimensions of right matrix or the multiplier must be equal to the column dimension of the left one. The row dimension of result matrix is equal to the left row dimension while the column dimension of result matrix is equal to the multiplier column dimension. Consider

(4) c[1,j]=(a[1,1]×  b[1,j])+a[1,2]×b[2,j]+a[1,3]×b[3,j],

(5) c[2,j]=(a[2,1]×  b[1,j])+a[2,2]×b[2,j]+a[2,3]×b[3,j],

(6) c[3,j]=(a[3,1]×  b[1,j])+a[3,2]×b[2,j]+a[3,3]×b[3,j].

Consider the later multiplication with constant values. The procedure is multiplying constant number to every element of the input matrix. The result matrix dimension will be equal to the input matrix dimension. Each element value in the result matrix can be computed from (7). These calculations are independent, and so the parallelism can be achieved as shown in Algorithm 3. Consider

(7) $$\begin{array}{c} c\left[i,j\right]=a\left[i,j\right]\times b. \end{array}$$

(*3) Parallel Division*. The matrix division calculation can be also performed in parallel because each calculation step is independent. The result at one position can be retrieved by dividing the same position of input matrix by constant values stated in (8) leading to an equalized dimensional matrix and the algorithm of parallel matrix division manipulation is illustrated in Algorithm 3. Consider

(8) $$\begin{array}{c} c\left[i,j\right]=\frac{a\left[i,j\right]}{b}\;. \end{array}$$

(*4) Parallel Transpose*. The matrix transpose procedure is to convert the rows into columns. For instance, the 1st row of input matrix becomes the 1st column of result matrix and the 2nd row of input matrix becomes the 2nd column of result matrix as shown in (9).

Example: 3 × 3 dimension matrix transpose:

(9) $$\begin{array}{c} {\left[\begin{array}{ccc} a_{1,1} & a_{1,2} & a_{1,3}\\ a_{2,1} & a_{2,2} & a_{2,3}\\ a_{3,1} & a_{3,2} & a_{3,3} \end{array}\right]}^{T}=\left[\begin{array}{ccc} c_{1,1} & c_{1,2} & c_{1,3}\\ c_{2,1} & c_{2,2} & c_{2,3}\\ c_{3,1} & c_{3,2} & c_{3,3} \end{array}\right]=\left[\begin{array}{ccc} a_{1,1} & a_{2,1} & a_{3,1}\\ a_{1,2} & a_{2,2} & a_{3,2}\\ a_{1,3} & a_{2,3} & a_{3,3} \end{array}\right]. \end{array}$$

Since the transformation from row to column is independent of each other, the parallelism can be utilized by using (10). The parallel matrix transpose manipulation algorithm is explained in Algorithm 2. Consider

(10) $$\begin{array}{c} {c\left[j,i\right]=a\left[i,j\right]}^{T}=a\left[j,i\right]. \end{array}$$

(*5) Parallel Transpose Multiplication*. For the matrix with symmetric and square property, the multiplication of this matrix with its transpose can be performed in parallel. Due to the nature of computational matrix in EM-PCA, the square matrix, one of which is the matrix multiplication with its transpose, *A* × *A* ^*T*^, and the calculation process could be optimized as stated in Algorithm 4 as follows: first, for diagonal computational elements, traditionally, the computation can be derived as stated in (11). However, the original matrix could be reused by removing the transpose process, for example, accessing and reserving memory, but performing the square of the original matrix instead:

(11) $$\begin{array}{c} \text{Diag}\left[i,j\right]=A{\left[i,j\right]}^{2}. \end{array}$$

Second, since the matrix is symmetry, each element in the upper-triangular matrix is the same as that in the lower-triangular matrix. (see (13)) leading to lower computational complexity by half (see (12)). Consider

(12) $$\begin{array}{c} \text{Upper}=\text{Lower}=\overset{n}{\underset{i=0}{\sum }}\overset{n}{\underset{j=0}{\sum }}a\left[i,j\right]. \end{array}$$

Optimized matrix multiplication with its transpose (upper/lower and diagonal). Consider

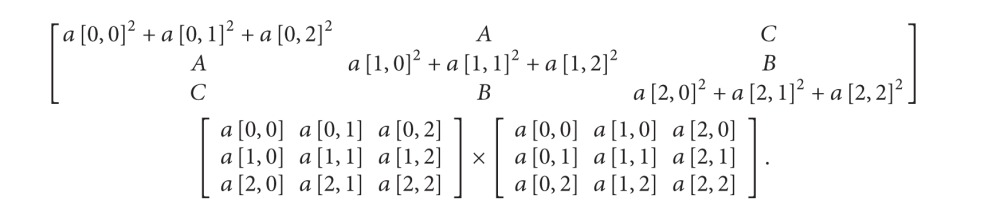
(13)


(*6) Parallel Determinant and Cofactor*. The cofactor matrix of *A* is the *n* × *n* matrix *C* whose (*i*, *j*) entry is the (*i*, *j*) cofactor of *A* (see (14)):

(14) $$\begin{array}{c} C_{ij}={\left(-1\right)}^{i+j}A_{ij}. \end{array}$$

The calculation of each position can be computed at the same time, and so the parallelism can be utilized as shown in (15).

Example: 3 × 3 dimension matrix cofactor calculation:

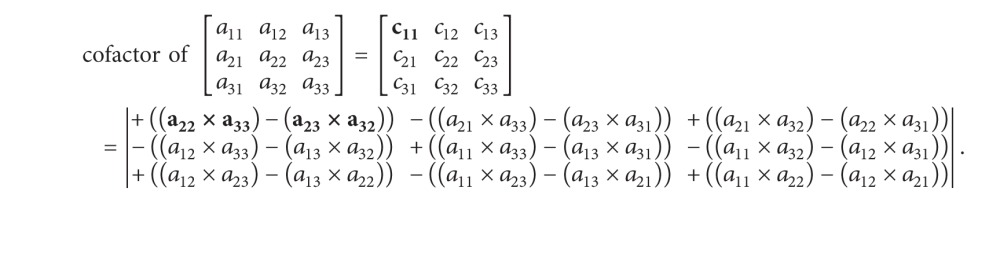
(15)


It should be noted that the determinant is a function of a square matrix reduced into a single number. Finding the determinant of an *n*-square matrix for *n* > 2 can be done by recursively deleting rows and columns to create successive smaller matrices until they are all in 2 × 2 dimensions.

Given the *n* × *n* matrix (*a*_*i*,*j*_), the determinant of *A* can be written as the sum of the cofactors of any rows or columns of the matrix multiplied by the entries that generated them. In other words, the cofactor expansion along the *j*th column gives

(16) det⁡(A)=a1jC1j+a2jC2j+  a3jC3j+⋯+anjCnj=∑i=1naijCij.

The cofactor expansion along the* i*th row gives

(17) det⁡(A)=ai1Ci1+ai2Ci2+  ai3Ci3+⋯+ainCin=∑j=1naijCij.

From (16) and (17), it is noticeable that their summation can be also in parallel. As shown in (18), the determinant value at each position can be computed at the same time because the calculation was independent. Both determinant and cofactor can be calculated concurrently as shown in Algorithm 5, and here, the calculation is performed iteratively until the matrix size equals 2 × 2 matrix.

**Figure 6:**
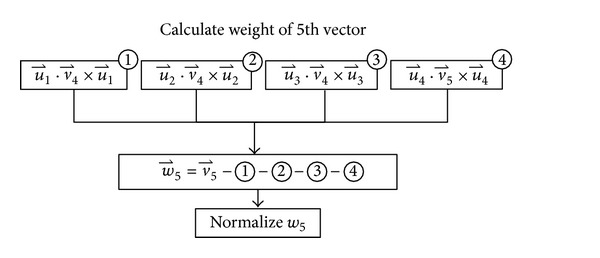
Example: weight calculation of the 5th vector.

Example: 3 × 3 dimension matrix determinant calculation:

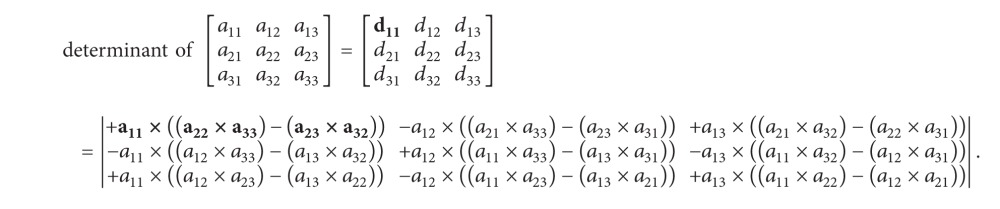
(18)


(*7) Parallel Orthogonal*. Gram-Schmidt orthonormalization is a method for converting a set of vectors into a set of orthonormal vectors. It basically begins by normalizing the first vector and iteratively calculating a weight vector of the remaining vectors and then normalizing them. The matrix can be normalized by first power every matrix component by two, then summarizes the result in each column, and finally divides each matrix component in each column by the square root of the summary. Notice that the orthogonalization processes proposed in most of the previous works [29] performed modified Gram Schmidt; however, in this research, a traditional algorithm, classical Gram Schmidt [46], was selected in order to gain higher degree of parallelisms as shown in Figure 6 and Algorithm 6.

Algorithms 2 to 6 illustrate our proposed matrix manipulation calculation, and in general,* parallel for* was applied [44] in  .NET C# to achieve process concurrency. Each of the computational tasks running over the algorithmis parameterized by an iteration value. It should be noted that, during the actual implementation, to achieve the process concurrency, there is a possibility to generate out-of-bound index. In this case, we applied a delegate function to obscure this problem over resource allocation. In this proposal,  .NET C# was implemented instead of MATLAB due to the limitation of computational matrix for huge dimensions over memory management. Most importantly, in addition, this implementation is suitable for online-processing face recognition.

### 4.3. Parallel Face Classification

**Figure 7:**
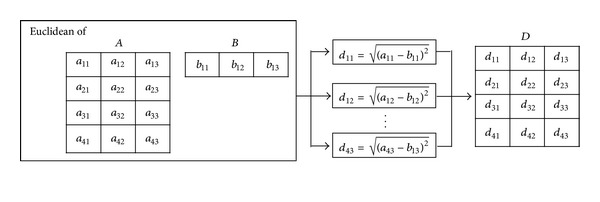
Parallel matrix operation computation for generalized Euclidean distance.

With our proposed parallel architecture face recognition, for testing purposes, our parallel classification is based on ED to parallelly figure out the closet distance of face [9] for simplicity purposes while remaining the recognition precision, especially during the matrix computation. Traditionally, one of the optimization techniques proposed in this research is based on Li et al. [43] by utilizing the input matrix characteristic (one-dimensional scale) leading to complexity reduction of (*οn* ^3^) to (*οn* ^2^) as shown in Algorithm 7. Furthermore, to utilize the parallelism for our parallel face recognition architecture, Figure 7 shows the feasibility to utilize the degree of parallelisms over our optimized ED in each ED's matrixes. The matrixes will be simultaneously computed in parallel; for example, with four processes, the computation at *d*_11_, *d*_12_, *d*_13_, and *d*_21_ will be performed in parallel. It should be noted that other probable classification techniques can also be fit in this architecture with paralleled modification.

## 5. Performance Evaluation

To investigate the performance of our parallel architecture, in this section, this research comparatively evaluates the system into three main scenarios in order to illustrate the optimal number of eigenvectors and epsilon values over EM-based approaches, to state the recognition computational complexity and precision, and to evaluate the performance of degree of parallelization.

### 5.1. Experimental Setup

In general, our testbed is based on a standard configuration on Windows 7 Ultimate operating systems (64 bits): CPU Intel(R) Core (TM) i-3770K 8-Cores 3.50 GHz (8 MB L3 Cache), 8192 × 2 MB DDR3-SDAM, and 500 GB 5400 RPM Disk.

The performance evaluation process was implemented in  .NET C# programming environment in order to emulate the real-world application and illustrate the computational time complexity. Public face database from FACE94 and FACE95 [47] was used for testing purposes. A set of colors was selected as 24-bits RGB, PNG images, ranging from 100 to 500 images of 180 × 200 pixels. In all evaluations, there are two main metrics: average (computational time and accuracy) and standard deviation for varied random loads (a number of trained images) over five trails [9]. Note that each evaluation, a classic statistical method was selected, that is, simple random selection [48], by randomly selecting nine testing images, four images within the training image dataset and five from the outside.

Three main scenarios are as follows: first, to illustrate the optimal number of eigenvectors and epsilon values for face recognition using EM-PCA and PEM-PCA in practice, the number of eigenvectors was varied by factor of 0.01, 0.03, 0.05, 0.07, and 0.09, respectively. In addition, various epsilon values, 1.0*E* − *x* in that *x* is in range of 1, 3, 5, 7, and 9, respectively, were applied in order to figure out the optimal value yielding the performance improvement over computational time, and in this scenario, the number of training images was limited to 500 [47].

Second, the selected optimal number of epsilons and eigenvectors are based on the outstanding performance in the first scenario. Then, for scalability purposes in terms of number of images, to illustrate the outstanding performance of our PEM-PCA, the evaluation was to comparatively perform the recognition process over two matrixes. Here, a traditional PCA, our first enhancement - EM-PCA, Parallel PCA (P-PCA), and finally our PEM-PCA. Note that the selected proposal, P-PCA is based on one of the promising previous works [35] in which divided the training dataset into modules regarding the number of CPU cores; that is, with eight cores, the dataset will be divided into eight sub-dataset, and then perform the recognition in parallel.

Finally, to evaluate the performance of degree of parallelization, especially of PEM-PCA including P-PCA and EM-PCA, the comparative performance over number of cores were in range of 1, 2, 4, and 8 cores, respectively. The computational time and accuracy were measured with 500 training images and 1.0*E* − 1 in epsilon and 0.009 in number of eigens.

### 5.2. Experimental Results

**Figure 8:**
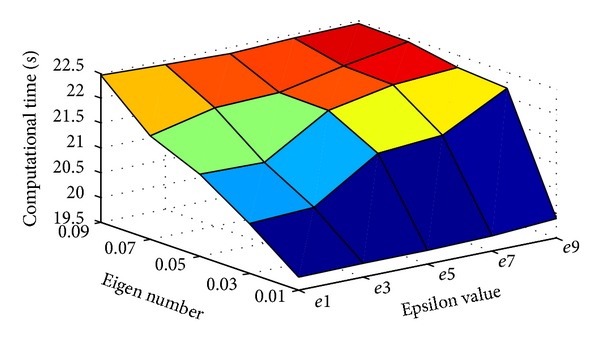
Effect of epsilon values and Eigen numbers on computational time over EM-PCA and PEM-PCA.

**Figure 9:**
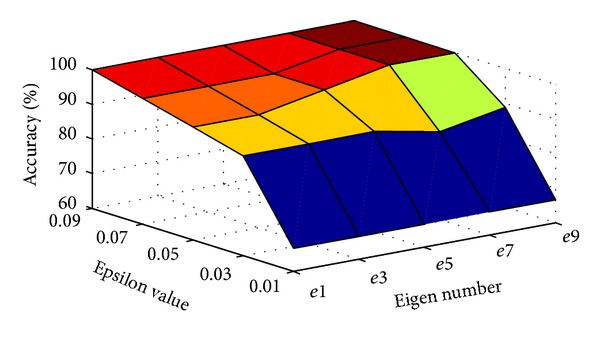
Effect of epsilon values and Eigen numbers on recognition accuracy over EM-PCA and PEM-PCA.

Consider the first scenario (EM-based approaches). Generally, Figure 8 illustrates that, by increasing a number of eigenvectors, the results indicated higher computational time-consuming operations. However, while increasing of epsilon values leads to lower computational time complexity. Similarly, consider the second scenario. Figure 9 shows the increasing trend of accuracy when the eigenvector number is increased. Nevertheless, the variation of epsilon value has insignificantly affected the recognition accuracy.

**Figure 10:**
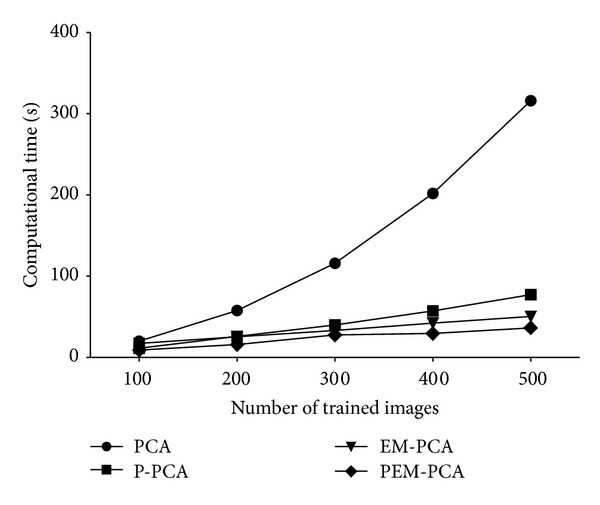
Computation time over number of trained images (PCA, P-PCA, EM-PCA, and PEM-PCA).

Second, to explicitly show the performance improvement of PEM-PCA, Figure 10 illustrates the computational speed, and especially with 500 images, PEM-PCA outperforms the other three approaches: PCA, P-PCA, and EM-PCA, by factor of nine, three, and two, respectively. In addition, by ranging the number of training images, the computational speed is in an increasing order. For example, with 100, 200, 300, and 400 images, the speed-up of PEM-PCA over the three approaches is in order of 3, 2, 2; 4, 2, 2; 5, 2, 2; 7, 2, 2, respectively. Note that the speed-up of the enhancement also depends on the degree of parallelisms and the number of cores as well as the number of working individual threads.

**Figure 11:**
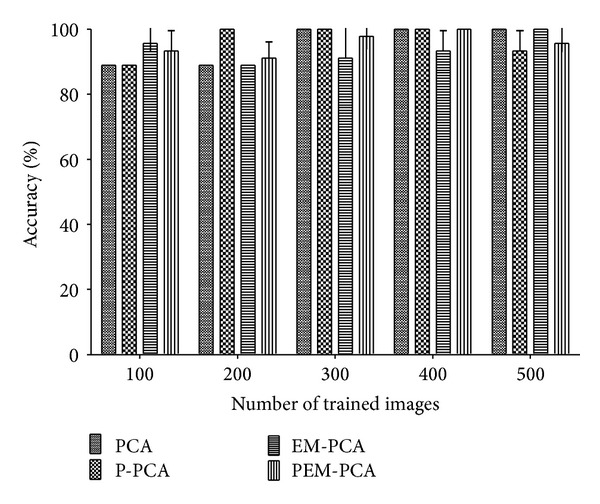
Percentage of recognition accuracy over number of trained images (PCA, P-PCA, EM-PCA, and PEM-PCA).

Moreover, consider the recognition precision. Figure 11 shows that the recognition accuracy of PEM-PCA is insignificantly different from the other three. It should be noted that this behavior of PEM-PCA and EM-PCA is due to the random characteristic when choosing the random eigenvectors.

**Figure 12:**
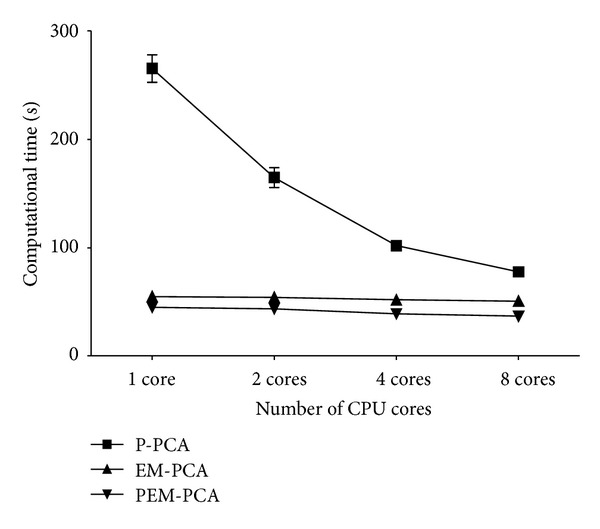
Computation time over number of CPU cores (P-PCA, EM-PCA, and PEM-PCA).

**Figure 13:**
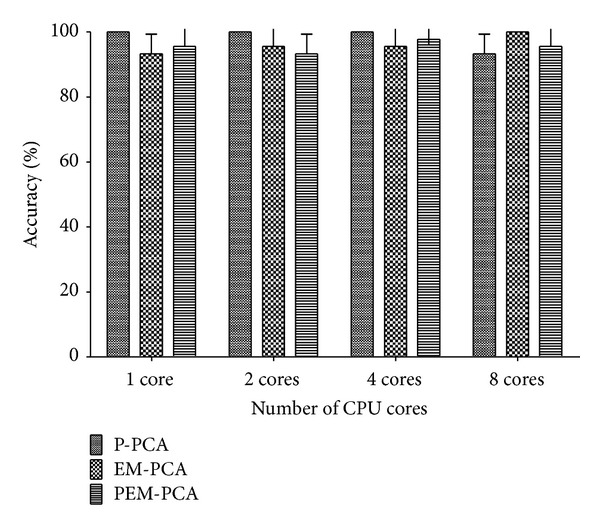
Percentage of recognition accuracy over number of CPU cores (P-PCA, EM-PCA, and PEM-PCA).

**Figure 14:**
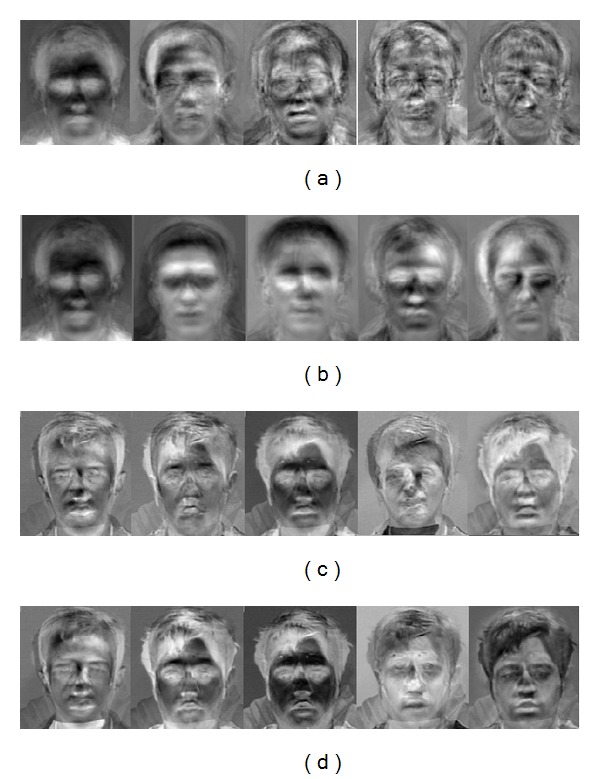
Eigenface decomposition ((a) traditional PCA, (b) P-PCA, (c) EM-PCA, and (d) PEM-PCA).

Finally, to illustrate the scalability when increasing the number of cores, Figure 12 shows that EM-PCA speed-up has no significant effect by the increase of cores, and especially with low number of cores, it incurs higher performance than P-PCA but not over PEM-PCA. Whenever the number of cores is accordingly increased, although P-PCA performance shows the degree of parallelism effect, PEM-PCA still has outstanding performance over the others, that is, in order of three and two of P-PCA and EM-PCA, respectively, at eight cores. In addition, similarly, P-PCA, EM-PCA, and PEM-PCA produced insignificant different face recognition precision as shown in Figure 13. Finally, in order to justify our algorithm implementation, Figure 14 shows comparative results of Eigenface decomposition among PCA, EM-PCA, P-PCA, and PEM-PCA.

## 6. Conclusions and Future Work

Although a traditional PCA can improve the recognition accuracy for a face recognition system, however, there exists a limitation over PCA. Therefore, in this research, several issues were evaluated and investigated, especially in terms of the computational time complexity during the covariance matrix computation stage. In addition, one of the possibilities to enhance PCA, Expectation-Maximization (EM) PCA face recognition, was proposed to enhance the time complexity when the recognition accuracy remains insignificant difference. Plus, due to the advance of parallelism, novel face recognition architecture was proposed by applying parallelism for large matrix manipulation including parallel preprocessing, parallel recognition, and parallel classification, all of which refer to Parallel Expectation-Maximization PCA or PEM-PCA.

Based on our justified parallel algorithm implementation, PEM-PCA outperforms the others, namely, a traditional PCA, our first enhancement, EM-PCA, and Parallel PCA by nine, two, and three, respectively. It should be noted that the results also depend on the number of training images with insignificant difference for recognition precision. Although the proposed technique can achieve a high degree of speed-up over PCA, more investigation including intensive evaluations can be performed, for example, improving preprocessing stages, enhancing high degree of parallelism aside from focusing only on matrix manipulation, reducing sensitivity outliers, and testing a large number of various images. In addition, to show the efficiency of parallelism usages, autonomously separating recognition task can be performed over message passing interface on each individual machine. To also fully complete the process of face recognition system, the other aspects of the system, that is, face detection, can also be further investigated. They are left for future work.

## Figures and Tables

**Algorithm 1:**
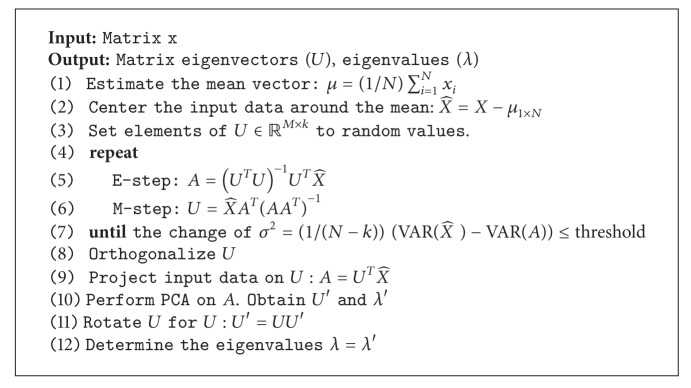
Expectation-Maximization algorithm.

**Algorithm 2:**
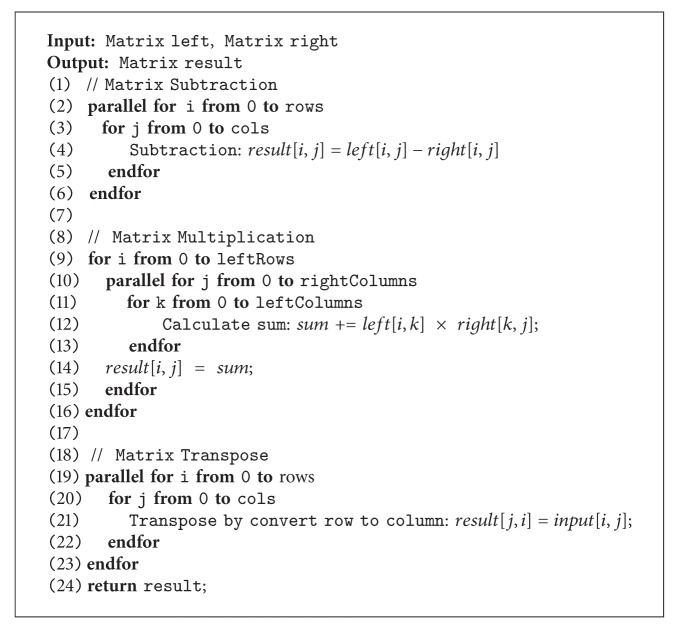
Parallel matrix manipulation with matrix computation.

**Algorithm 3:**
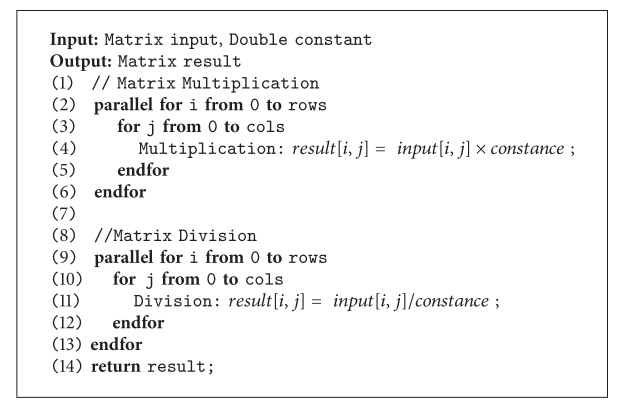
Parallel matrix multiplication with a constant number computation.

**Algorithm 4:**
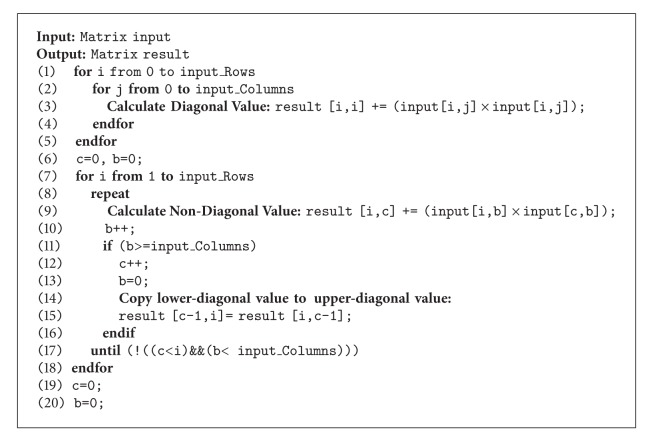
Parallel optimized matrix multiplication with its transpose algorithm [27].

**Algorithm 5:**
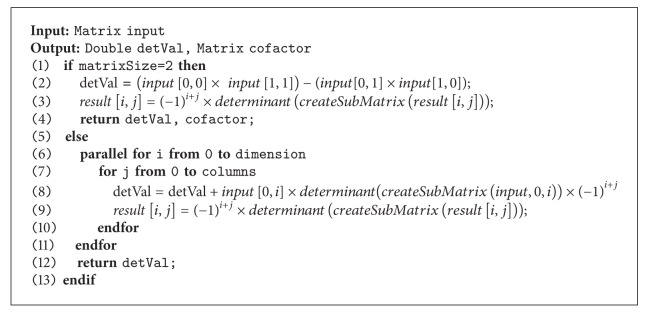
Parallel matrix determinant and cofactor computation.

**Algorithm 6:**
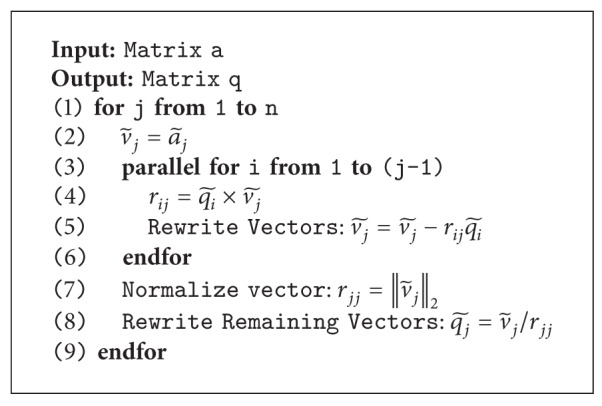
Proposed parallel matrix orthogonalization computation.

**Algorithm 7:**
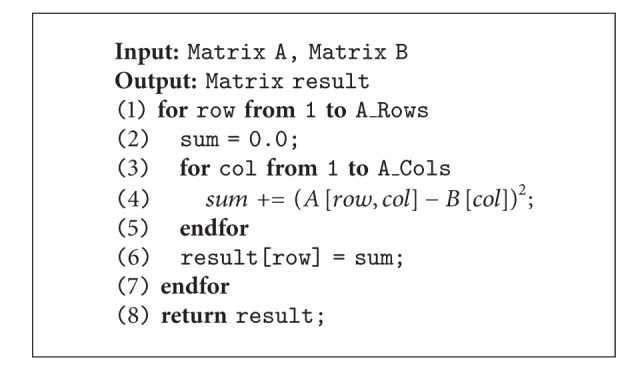
Matrix Operation for Generalized Euclidean Distance.
